# Interpretation of the Consequences of Mutations in Protein Kinases: Combined Use of Bioinformatics and Text Mining

**DOI:** 10.3389/fphys.2012.00323

**Published:** 2012-08-22

**Authors:** Jose M. G. Izarzugaza, Martin Krallinger, Alfonso Valencia

**Affiliations:** ^1^Structural Computational Biology Group, Structural Biology and BioComputing Programme, Spanish National Cancer Research CentreMadrid, Spain

**Keywords:** disease, kinase, literature mining, mutation, pathogenicity prediction, protein kinase, text mining, variation

## Abstract

Protein kinases play a crucial role in a plethora of significant physiological functions and a number of mutations in this superfamily have been reported in the literature to disrupt protein structure and/or function. Computational and experimental research aims to discover the mechanistic connection between mutations in protein kinases and disease with the final aim of predicting the consequences of mutations on protein function and the subsequent phenotypic alterations. In this article, we will review the possibilities and limitations of current computational methods for the prediction of the pathogenicity of mutations in the protein kinase superfamily. In particular we will focus on the problem of benchmarking the predictions with independent gold standard datasets. We will propose a pipeline for the curation of mutations automatically extracted from the literature. Since many of these mutations are not included in the databases that are commonly used to train the computational methods to predict the pathogenicity of protein kinase mutations we propose them to build a valuable gold standard dataset in the benchmarking of a number of these predictors. Finally, we will discuss how text mining approaches constitute a powerful tool for the interpretation of the consequences of mutations in the context of disease genome analysis with particular focus on cancer.

## The Human Kinome

Protein kinases are a family of enzymes that catalyze the transfer of a phosphate from ATP to a serine, threonine, or tyrosine hydroxyl group in the target protein. Phosphorylation often implies enzyme activation or inhibition, alteration of interaction surfaces, and conformational changes, among the most common consequences. It is due to the importance of the processes regulated, that protein kinases generally do not act alone but rather, they form part of a finely tuned signaling cascade that is strictly controlled spatiotemporally. Therefore, protein kinases are metaphorically referred to as the metabolic switches of the cell.

Protein kinases are one of the most ubiquitous families of signaling molecules in the human cell. The total number of genes encoding kinases has been a matter of discussion in the last decade and, for instance, in Wang (1998) estimated between 1000 and 2000 different human kinase genes. With the completion of the human genome, the current estimate is that 518 genes encode protein kinases, corresponding to more than 2% of the total number of genes in the human genome (Manning et al., 2002b).

All members of the superfamily share a characteristic domain – the protein kinase domain – that confers them the ability to phosphorylate other proteins. Empirical studies suggest that the residues conforming the ATP binding site tend to be conserved and that phosphotransfer is carried out by a shared set of amino acids (Schee and Bourne, 2005; Knight et al., 2007; López et al., 2007; Kinnings and Jackson, 2009; Tanramluk et al., 2009).

In spite of these similarities, experiments in yeast models (Manning et al., 2002a; Ubersax et al., 2003) suggest that although protein kinases individually present a remarkable substrate specificity, the superfamily as a whole is very promiscuous, phosphorylating a wide range of protein substrates. This observation, may be attributed to the different domain architectures present in the protein kinase superfamily. In addition to the aforementioned protein kinase domain committed to the general function of phosphorylation, a number of modular domains are combined to, for example, confer substrate specificity, to tightly control the activity of the enzyme or anchor the kinase to the membrane (Finn et al., 2010).

These differences in terms of functionality and domain architecture can be used to classify members of the protein kinase superfamily into different categories. Indeed, there are several different classifications of kinases from the main model organisms: yeast (Hunter and Plowman, 1997), worm (Manning, 2005), fruit fly (Manning et al., 2002a), and mouse (Caenepeel et al., 2004). The reference classification in humans is KinBase (Manning et al., 2002b; Miranda-Saavedra and Barton, 2007), which has also been incorporated into UniProt (Bairoch et al., 2005), albeit with minor modifications.

## Mutations in the Protein Kinase Superfamily

Due to their important regulatory function, a number of mutations in protein kinases have been associated with different human diseases (Shchemelinin et al., 2006), including cancer. For example, Greenman et al. (2007) carried out the first large scale study of the variation of 518 human kinases in 210 samples of cancer tissues and cell-lines. Moreover, other high-throughput studies (Sjöblom et al., 2006; Wood et al., 2007) also yielding interesting information about the role that variation of human protein kinases plays in cancer. For a detailed review, refer to Baudot et al. (2009).

The results from these high-throughput resequencing projects is often available through research publications. However, in order to make the information more easily accessible, several efforts are devoted to compile, store, annotate, and characterize mutations, including mutations in the protein kinase superfamily. Some examples are UniProt (Yip et al., 2008), COSMIC (Bamford et al., 2004), SAAPdb (Hurst et al., 2009), MoKCa (Richardson et al., 2009), and KinMutBase (Ortutay et al., 2005). Together they constitute a powerful resource to understand disease association and the functional/structural properties of the mutations that affect human protein kinases.

Unfortunately, database curators are not able to store and annotate the vast amount of information provided by large-scale variation studies at the same pace it is generated. Mainly, because the process generally involves the manual inspection and curation of specific variation studies, which requires considerable resources. As a consequence, although growing in number, the mutations totally characterized, and well-understood only represent a small fraction of all the human variome.

## Methods to Predict Pathogenic Mutations

In the section Mutations in the protein kinase superfamily we mentioned that high-throughput resequencing screenings represent a powerful set of techniques to discover large numbers of mutations. Of these, only a small fraction are causally implicated in disease onset and therefore, separating the wheat from the chaff is still a major challenge (Baudot et al., 2009). For a small subset of the new mutations discovered, experimental information is available regarding the relationship between the mutation and disease, and for a smaller number of cases the underlying biochemical mechanism is known. Little information is available for the remaining mutations. The requirement of a lot of investment, both in terms of time and money, means that it is not feasible to experimentally test the association of all these mutations to disease, and to characterize their functional effects. Nevertheless, this problem is very amenable to *in silico* predictors.

Cline and Karchin (2011) wisely summarized the two different approaches as follows: “*A bench biologist interested in whether a mutation of interest impacts the transcription of a gene might perform site-directed mutagenesis on genomic DNA, transfect mutated DNA into cell culture, and use readouts of the gene’s transcriptional activity to measure changes with respect to wild type. In contrast, a bioinformatics approach typically involves computational analysis of the DNA sequence surrounding the mutation, possibly supplemented with information from published bench experiments*.”

This is just one example of the very different methods available to predict *in silico* the probability of a newly discovered mutation being implicated in disease. Different approaches have been developed in the last decade (Table 1) and several detailed reviews on this subject have been published (Baudot et al., 2009; Karchin, 2009; Cline and Karchin, 2011).

**Table 1:** **Summary of methods to predict the pathogenicity of mutations**.

Method	Main features	Further information
SIFT (Ng and Henikoff, 2001)	Threshold-based, conservation	http://sift.jcvi.org
PMUT (Ferrer-Costa et al., 2005)	Neural Network, sequence-, and structure-based features	http://mmb.pcb.ub.es/PMut
SNPs3D (Yue et al., 2006)	Support Vector Machine, structure-based features	http://www.snps3d.org
PANTHER (Thomas et al., 2003)	Threshold-based, conservation (PSEC)	http://www.pantherdb.org/tools/csnpScoreForm.jsp
Pfam LogRE (Clifford et al., 2004)	Threshold-based, probability of a PFAM domain to be pathogenic using a log-odds ratio	
LS-SNP (Karchin, 2009)	Support Vector Machine, sequence-, and structure-based features	http://ls-snp.icm.jhu.edu/ls-snp-pdb
CanPredict (Kaminker et al., 2007a)	Combines SIFT, Pfam LogRE, and Gene Ontology terms in a single prediction	http://research-public.gene.com/Research/genentech/canpredict
SNAP (Bromberg and Rost, 2007)	Neural Network, sequence-, and structure-based features	http://cubic.bioc.columbia.edu/services/snap
Torkamani (Torkamani and Schork, 2007)	Support Vector Machine, sequence-, and structure-based features, kinase-specific	
MutaGeneSys (Stoyanovich and Pe’er, 2008)	Whole-genome marker correlation dataset to identify association to causal SNPs in OMIM	http://www.cs.columbia.edu/~jds1/MutaGeneSys
stSNP (Uzun et al., 2007)	Integrates non-synonymous SNPs from dbSNP, structural models from Modeler and KEGG pathways. Comparative native/mutant analysis	http://ilyinlab.org/StSNP
F-SNP (Lee and Shatkay, 2008)	Metaserver, combines PolyPhen, SNPeffect2.0, SNPs3D, LS-SNP	http://compbio.cs.queensu.ca/F-SNP
SNP & GO (Calabrese et al., 2009)	Support Vector Machine, several sequence-derived features, and information from Gene Ontology terms	http://snps-and-go.biocomp.unibo.it/snps-and-go/
PolyPhen-2 (Adzhubei et al., 2010)	Bayesian classifier, sequence-, and structure-based features	http://genetics.bwh.harvard.edu/pph2
MuD (Wainreb et al., 2010)	Random forest, sequence-, and structure-based features	http://mud.tau.ac.il
CHASM (Wong et al., 2011)	Random forest, sequence-based features	http://wiki.chasmsoftware.org/index.php
Mutation Assessor (Reva et al., 2011)	Threshold-based, differential evolutionary conservation in subfamilies	http://mutationassessor.org
Condel (González-Pérez and López-Bigas, 2011)	Metaserver, combines the output of other predictors	http://bg.upf.edu/condel/
wKinMut (Izarzugaza et al., 2012, submitted)	Framework for the analysis of kinase mutations. Integrates annotations, predictions, and information from the literature	http://wkinmut.bioinfo.cnio.es

These methodologies can be classified according to their underlying principles: Some methods make use of several features to identify relevant positions in a given protein, and hence, rules are derived to predict the pathogenicity of mutations. Another group of implementations assumes that evolutionarily conserved protein residues are important for protein structure, folding, and function, whereby mutations in these residues are considered deleterious (Ng and Henikoff, 2001). Variations on this principle lead to methods that predict deleterious mutations by assessing the changes in evolutionarily conserved PFAM motifs (Clifford et al., 2004). Furthermore, a group of methodologies use protein structures to characterize substitutions that significantly destabilize the folded state. A growing number of systems integrate prior knowledge in the form of both sequence-based and structure-based features from a set of mutations (for which their characterization as pathogenic or neutral exists) to train an automatic machine learning system. Once trained, the system can infer the pathogenicity of new mutations automatically. Different machine learning methods can be implemented depending on their individual needs. Among them, probably the most popular ones are: rule-based systems (Wang and Moult, 2001; Ramensky et al., 2002; Reva et al., 2011), decision trees (Krishnan and Westhead, 2003), random forests (Kaminker et al., 2007b; Wainreb et al., 2010), neural networks (Ferrer-Costa et al., 2002; Bromberg and Rost, 2007), Bayesian methods (Adzhubei et al., 2010), and SVMs (Karchin et al., 2005; Yue et al., 2005; Torkamani and Schork, 2007; Calabrese et al., 2009; Wainreb et al., 2010). In addition, some meta approaches have been implemented recently (Lee and Shatkay, 2008), for instance, Condel (González-Pérez and López-Bigas, 2011) integrates five of the most widely employed computational tools for sorting missense single nucleotide variations.

Methods also differ in the nature of the protein properties used to determine the pathogenicity of new mutations. Some of the predictors require sequence-oriented features that are easily applicable to any polymorphism. Recurrent examples of this category are: amino acid type, sequence conservation, domain type, functional annotations, post-translational modifications, and so on. A second set of predictors calculate features that require a protein structure. Common examples to illustrate these are: secondary structure, solvent accessibility, flexibility, etc. The major drawback of these methodologies is that although they may increase the accuracy, the need for either an experimentally solved or a precisely modeled protein structure implies a loss of coverage. The number of features and their combinations is infinite. Moreover, features can also either be general or apply only to a defined subset of proteins, as is the membership to a kinase group (Torkamani and Schork, 2007; Izarzugaza et al., 2012).

## Benchmarking Prediction Methods

In the previous section we discussed the differences between the various methods, both in terms of implementation and prediction features. Equally important are the differences found in the composition of the datasets used to train the methods. This is particularly relevant in the case of machine learning approaches. Machine learning approaches are developed in two independent consecutive steps: during the initial development phase, the developers aim to optimize the combination of features, internal parameters, and prediction algorithms to obtain a trained classifier. In a later phase, blind tests are conducted to evaluate the performance simulating a more realistic scenario. Consequently, three separate datasets are needed: (i) a training dataset to allow the classifier to learn, (ii) a validation dataset to optimize the selection of parameters, and (iii) an evaluation dataset to conduct blind tests to assess the expected performance of the classifier.

Consequently, the datasets used highly influence the overall performance of the prediction and, if not pondered cautiously might become a source of evaluation errors. Probably, the most common of them being overtraining as a result from the evaluation of the methodologies with mutations that have also been considered in the training dataset. In other words, if a predictor were evaluated using a test set whose correct answers the method had previously been provided with, this may yield unfair over-estimation of the prediction capability. An extension of this problem, especially if the features considered predict at the protein level, is that mutations occurring in the same protein or closely related homologs should not span two different datasets.

The selection of a benchmark dataset that is fair and does not lead to artifacts is not a trivial task (Care et al., 2007) and clean datasets that were not used in the development of any of the methods are required. Following a similar approach to those in the detection of bio-entities from the literature (BioCreative), protein structure (CASP), and protein interaction prediction (CAPRI), a successful recent example is CAGI^1^. In summary, CAGI is intended to assess a battery of computational methods for predicting the phenotypic impacts of genome variation. Participants are provided a number of different sets of genetic variants and are expected to make predictions of resulting, molecular, cellular, or organismal phenotype. These predictions are later on evaluated by independent assessors against experimental characterizations.

Although CAGI constitutes an undoubtedly powerful tool to provide insights on the performance of state-of-the-art methodologies, the major drawback is that provided datasets are gathered from very specialized projects, and consequently are seldom universally applicably to all methodologies, which consequently, limits the benchmark. An example of the previous would be the intrinsic limitation to predict mutations outside the protein kinase superfamily for kinase-specific methodologies.

Complementary to the CAGI experiment, current text mining methodologies enable the generation of clean sets of experimentally validated mutation mentions from the literature. Those mutations that were not recorded in the databases used to provide the training and evaluation datasets are of special interest. Here we propose a pipeline for the curation of mutations automatically extracted from the literature and their use as a gold standard in the benchmarking of pathogenicity predictors. We will describe this approach thoroughly in the following sections.

## Mining Kinase Mutations from the Literature

Previously, we discussed how the efforts of database curators to store and annotate mutations (Table 2) can hardly keep the pace of the vast amount of information generated by current large-scale variation studies. To bridge this growing gap, automatic extraction of entities and their relationships from the existing literature can be applied. This includes text mining techniques such as regular expressions, pattern recognition, and natural language processing, among others. Indeed, these approaches have been successfully applied to other fields of research, for instance for the automatic extraction of protein–protein interactions (Blaschke and Valencia, 2001; Krallinger et al., 2008c) and in the annotation of genes and proteins (Krallinger et al., 2008a, 2010). Despite the success of these methods, it must be born in mind that this technology does not aim to replace manual curation and validation. Rather, text mining approaches are better understood as systematic tools to assist the efforts of human curators by helping them to find information, prioritize documents, and highlight potentially relevant items (Krallinger et al., 2008a,b; Leitner et al., 2010).

**Table 2:** **Summary of resources providing information about kinases and mutations**.

Method	Description	Further information
UniProt (Consortium, 2007)	General information about proteins, including human protein kinases	http://www.uniprot.org/
PDB (Berman et al., 2000)	Catalog of protein structures, protein kinases widely represented	http://www.rcsb.org/
PDBsum (Laskowski et al., 2005)	Annotation on protein structures	http://www.ebi.ac.uk/pdbsum
KinBase (Manning et al., 2002b; Miranda-Saavedra and Barton, 2007)	Hierarchical classification of protein kinases	http://kinase.com/kinbase/
SwissVar (Yip et al., 2007)	Detailed information about mutations present in UniProt	http://swissvar.expasy.org/
COSMIC (Bamford et al., 2004)	Catalog of somatic mutations in cancer	http://www.sanger.ac.uk/perl/genetics/CGP/cosmic
Ensembl (Flicek et al., 2011)	Infrastructure for the integrated annotation on chordate and selected eukaryotic genomes	http://www.ensembl.org
dbSNP (Sherry et al., 2001)	Annotated catalog of SNPs	http://www.ncbi.nlm.nih.gov/projects/SNP
HapMap (Consortium et al., 2010b)	Catalog of common genetic variants in the human genome	www.hapmap.org
1000 Genomes (Consortium et al., 2010c)	Deep catalog of human variations derived from the next-generation sequencing of 1000 people	http://www.1000genomes.org/
TCGA (Network, 2011)	The Cancer Genome Atlas is a collection of genetic variations found in 20 different cancers	http://cancergenome.nih.gov/
ICGC (Consortium et al., 2010a)	The International Cancer Genome Consortium project aims to a comprehensive description of genomic, transcriptomic, and epigenomic changes in 50 tumor types and sub-types	http://www.icgc.org
OMIM (Amberger et al., 2011)	Catalog of Mendelian mutations known to cause disease	http://www.ncbi.nlm.nih.gov/omim
SAAPdb (Hurst et al., 2009)	Calculation of the structural consequences of mutations	http://www.bioinf.org.uk/saap/db/
SNPeffect 2.0 (Reumers et al., 2006)	A database mapping molecular phenotypic effects of human non-synonymous coding SNPs	http://snpeffect.switchlab.org
ModBase (Pieper et al., 2006)	Structural models of mutant proteins	http://salilab.org/modbase
TopoSNP (Stitziel et al., 2004)	TopoSNP: a topographic database of non-synonymous single nucleotide polymorphisms with and without known disease association	http://gila.bioengr.uic.edu/snp/toposnp/
MoKCa (Richardson et al., 2009)	Annotated catalog of cancer-associated mutations in protein kinases	http://strubiol.icr.ac.uk/extra/mokca/
KinMutBase (Ortutay et al., 2005)	Registry of disease-causing mutations in protein kinase domains	http://bioinf.uta.fi/KinMutBase

**Figure 1:**
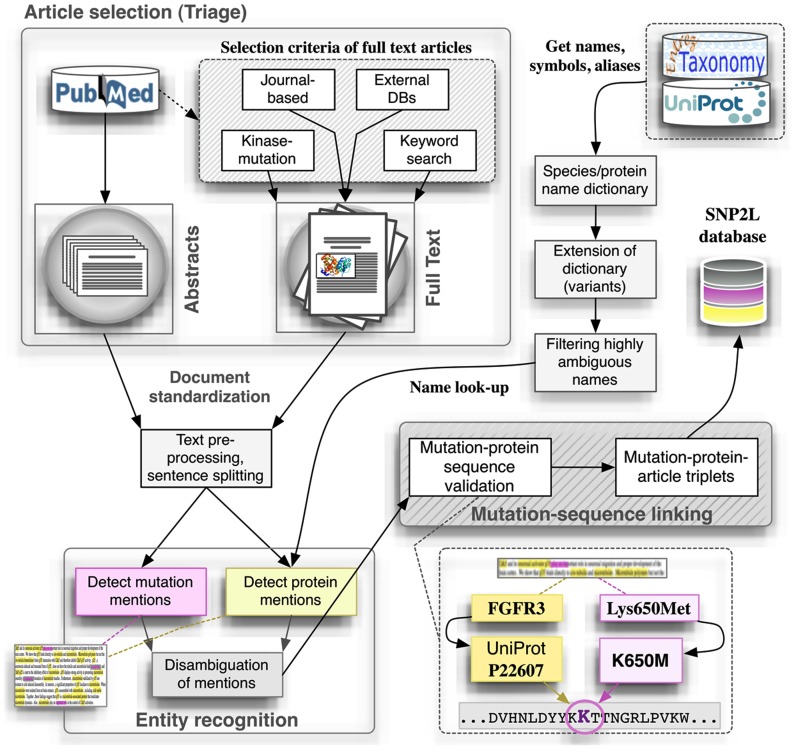
**SNP2L Pipeline as an example of a typical automatic method to extract mutation mentions from the literature**. The pipeline integrates article retrieval, detection of mutations and proteins in the corresponding article, correct mutation-protein association and, finally, validation of the results.

Here we will use our recently published pipeline for extracting mutation mentions in protein kinases from the literature, SNP2L (Krallinger et al., 2009), as an example of a typical text mining workflow. The pipeline (Figure 1) integrates article retrieval, detection of mutations, and proteins in the corresponding article, correct mutation-protein association and, finally, validation of the results. To the best of our knowledge there is currently no pipeline similar to the one presented here. Two main aspects make our pipeline unique. First, our system is specifically designed to extract mutations occurring in the protein kinase superfamily. Second, we perform an additional filtering step to ensure the quality of the extracted mutations as we will disclose in the following sections.

### Article selection (triage): Constructing a text mining corpus

Following a common approach in text mining, we tested SNP2L with two different datasets: One constituted by the whole collection of PubMed abstracts and the other by a collection of either manually or automatically selected full-text articles. In order to construct the *corpus*, full-text articles were automatically downloaded using an in-house retrieval system (Krallinger et al., 2008a) prioritized under three different criteria:

1. Relevance of the abstract: information contained in the corresponding abstracts such as the mention of mutations, mention of human kinases, and a combination of keywords (including “human kinase mutation”).
2. *A priori* relevance of the full-text articles: extracting all references in PubMed for human kinases contained in multiple databases (e.g., SwissProt, MINT, and IntAct).
3. Relevance of the journal: based on analyzing a fraction of mutation-mentioning abstracts of each journal and prioritizing a set of journals (and thus their articles) to retrieve their full-text articles. This set consisted of the following journals: *American Journal of Human Genetics, European Journal of Human Genetics, Human Genetics, Human Mutation, and Human Molecular Genetics*.

Before proceeding to the next step, all articles should be split in sentences using a sentence boundary detection system (Krallinger et al., 2008a).

### Entity recognition: Mutations and protein kinases

The consistent nomenclature used to describe mutations in the literature makes these entities especially amenable to this type of approach and accordingly, a growing number of such methods have been described in the literature over the years. A summary of several of these literature mining tools to extract information on mutations is presented in Table 3. In the example discussed here, we used MutationFinder (Caporaso et al., 2007) for the initial extraction of single aminoacid substitutions. MutationFinder constitutes a valuable tool to detect the mention of mutations in a given set of manuscripts and it relies on language expressions used to describe mutation events. MutationFinder is very competitive for recall and precision when compared to other strategies (Yip et al., 2007), and it has been evaluated using a manually generated gold standard collection of abstracts.

**Table 3:** **Summary of text mining implementations for mutation extraction**.

Method	Main features
MEMA (Rebholz-Schuhmann et al., 2004)	Regular expressions, gene and protein mentions, co-mention proximity, OMIM validation
MuteXt (Horn et al., 2004)	Regular expressions, GPCR and NR mentions detection, co-mention proximity, sequence check
Yip (Yip et al., 2007)	Regular expressions, protein mentions detection, SwissProt validation, sequence check
Mutation GraB (Lee et al., 2007)	Regular expressions, protein mentions detection, graph shorted distance, sequence check
Mutation Miner (Baker and Rene, 2006)	Regular expressions, protein mentions detection, sentence co-mention
MuGeX (Erdogmus and Sezerman, 2007)	Regular expressions, protein mentions, protein, and DNA mutation disambiguation
VTag (McDonald et al., 2004)	Machine learning detection of acquired sequence variation mentions detection (mutations, translocations, and deletions)
OSIRIS (Furlong et al., 2008)	Detection of human gene variations corresponding to SNPs
MutationFinder (Caporaso et al., 2007)	Regular expressions and patterns, protein mutations mentions detection, complex language expressions

After recognizing all the mutations mentioned in the text, we attempted to identify all human protein kinases co-mentioned with them in the same document. Existing systems that try to link mentions of genes and proteins to database identifiers generally rely on approaches that compare the names appearing in the text to gene names or aliases contained in database records. The actual task of determining the exact database record for a gene/protein mention is commonly referred to as gene mention grounding or normalization, and has been evaluated in the second BioCreative community challenge, illustrating that dictionary look-up approaches can obtain competitive results for this purpose (Morgan et al., 2008).

Following this line, we constructed a lexicon specifically for human protein kinases, derived from gene and protein symbols, names, and aliases contained in the UniProt database (see Figure 1, Get names, symbols, and aliases). Because this gene/protein lexicon did not capture all representative typographical variants of a given name, we used a rule-based approach and heuristics for generating typographical variants for the kinase lexicon entries. With this respect, the alternative use of hyphens, capitalization (upper-case and capitalized names), and different word order variants were captured. The gene/protein lexicon was filtered to eliminate highly ambiguous names through comparison with a stop word list and by, after an initial look-up step, checking manually potential outlier names that show a very high mention frequency. The extended and pruned human kinase lexicon was then used for the detection of corresponding mentions in our document collections containing mutation mentions. As a given name can correspond to different records (ambiguity), both at the level of human genes as well as in case of genes from different species sharing the same name, we calculated for each article, two different scores reflecting (a) the contextual similarity of the article to the reference (UniProt) protein record and (b) the overall association of the article to human species terms from the total set of tagged species terms. A conceivable alternative would be to simply apply very strict protein-organism co-mention criteria based on relative textual distances, which is rather problematic in case of human proteins were often the organism source is not explicitly stated.

### Mutation-sequence linking

The next step is to link mutation mentions with their corresponding human kinases. This step would be trivial if a single protein was mentioned per article, however, for most of the articles this is not the case and more than one protein is mentioned per article. A reasonable solution would be to check the existence of the amino acid at the specified position for each mutation mention-protein combination. In addition to this basic sequence look-up validation method additional mutation mapping strategies could be implemented. They should consider errors resulting from the wrong detection of the directionality of the extracted mutation mention (using the wild type as mutant residue and *vice versa*) and inconsistencies and alternative sequence counting between the article and the kinase sequence. For example:

– Sliding window algorithms that look for relative positions of mutations (pattern) rather than exact position co-occurrences. With this approach, mutation mentions would be scanned looking for positions relative to the starting one attending to the distance between all the mutations in the same abstract. The strength of this approach is that it is able to deal with alternative sequence coordinates. There are many examples in the literature: Mutations F175P, R178L, and Y530L in the proto-oncogene tyrosine-protein kinase Src, are mentioned in the considered article (PMID 2108315) as F172P, R175L, and Y527F respectively. Since the probability of finding simple patterns by chance can be high in some trivial cases, it is reasonable to consider only those cases where a minimum number of mutated positions (3 in our example) could be detected.
– Bidirectional mutation to sequence position mapping. Either the wild type or the mutant residue of an extracted mutation mention might be accepted in the corresponding sequence position.
– Pro-peptides and mature protein mutation mapping. In order to allow alternative residue counting due to the presence of a signal peptide, a displacement equal to the length of the corresponding signal peptide might be allowed.
– Methionine cleavage: the mutation mapping might be carried out taking into consideration the possibility of neglecting the N-terminal methionine.

### Using the literature to generate a benchmark dataset

The main focus of this article has been the construction of a gold standard dataset to benchmark prediction methods. Following this thread of reasoning, mutations already present in common databases are discarded, while new ones form the benchmark dataset. This procedure will ensure a dataset that enables fair comparison and is less prone to over-estimation of the classifiers’ performance as we discussed previously in the *Benchmarking prediction methods* section.

In spite of constituting a powerful tool for the extraction of knowledge from the literature, text mining approaches to recover kinase mutations still have some limitations in terms of recall and a number mutations escape detection by even the most accurate state-of-the-art algorithms. Among the challenging aspects in this respect are the detection of mutations that are described in additional materials or contained in tables and figures. This is because they can not easily be converted efficiently to plain text. Another key issue is the appropriate detection of the kinase mentions, which can be referred to through a range of different typographical variations and aliases, of which text mining approaches can only cover some. To this issue one also needs to add the underlying limitations in terms of recall of the mutation extraction process (Caporaso et al., 2007) and inconsistencies of sequence descriptions in reference databases as compared to those examined in scientific articles.

### Using the literature to understand the consequences of mutation

From a parallel perspective, text mining approaches can be used to enhance our understanding of both new and existing mutations. Text mining approaches output mutations extracted from the literature along with all their contextual information. Pointers to the relevant literature are provided, these include: experimental conditions, organism, or population sub-types, information regarding observed phenotypes including association to disease, or in a best case scenario, the underlying biochemical mechanisms.

This information can help to interpret the consequences of mutations and is often complementary to the valuable clues provided by the methods to predict the pathogenicity of mutations. Indeed, the emerging trend in the field is to integrate information from diverse sources (Lee and Shatkay, 2008; González-Pérez and López-Bigas, 2011), as we have done recently with the development of wKinMut^2^ to help in the interpretation of mutations in the protein kinase superfamily.

In addition to the predictions of pathogenicity directly from our in-house classifier (Izarzugaza et al., 2012) and the values of the features used in the classification, wKinMut combines information from different external sources to help in the interpretation of the prediction. These include the results from other classifiers focusing on different aspects of mutation pathogenicity (SIFT; Ng and Henikoff, 2001; MutationAssessor; Reva et al., 2011), the representation of the mutation in the context of its three-dimensional structure and records of the mutation in other databases such as SAAPdb (Hurst et al., 2009), UniProt (Yip et al., 2007), COSMIC (Bamford et al., 2004), and KinMutBase (Ortutay et al., 2005). Two text mining resources complement the framework: iHop (Hoffmann and Valencia, 2005) a literature mining system to extract gene–gene and protein–protein interactions and SNP2L (Krallinger et al., 2009) whose capabilities to detect mutation mentions from the literature have been described thoroughly here.

In summary, wKinMut can be useful to predict the pathogenicity of novel mutations and to interpret the biochemical mechanisms leading to pathogenicity and it can be applied to the interpretation of genomes from cancer patients.

## Overview and Summary

Current research aims to discover the mechanistic connection between mutations and disease. We focused on the protein kinase superfamily due to the enormous wealth of mentions in the literature associating different diseases, including cancer, with mutations in members of this superfamily.

In this article we have reviewed the different possibilities and limitations of state-of-the-art computational methods for the prediction of the pathogenicity of mutations and we have discussed the difficulties that arise to benchmark and evaluate the performance of the classifiers. We have proposed our recently published pipeline, SNP2L, for the automatic extraction and curation of mentions in the literature to collect a gold standard dataset that might be used in the benchmarking of the different predictors. Finally, we have introduced wKinMut as an example the integration of text mining with prediction methodologies to help in the interpretation of the consequences of mutations in the context of disease genome analysis with particular focus on cancer. We think that such applications might be of interest in the interpretation of patient genomes in the emerging field of personalized/stratified medicine in, hopefully, a near future.

## Conflict of Interest Statement

The authors declare that the research was conducted in the absence of any commercial or financial relationships that could be construed as a potential conflict of interest.

## Footnotes

^1^http://genomeinterpretation.org

^2^http://wkinmut.bioinfo.cnio.es

## Research Topic:

From structural to molecular systems biology: experimental and computational approaches to unravel mechanisms of kinase activity regulation in cancer and neurodegeneration

## Topic Editor:

Matteo Barberis, Humboldt University Berlin, Germany; Max Planck Institute for Molecular Genetics, Berlin, Germany
